# The Electrocution of Czolgosz

**Published:** 1901-11-30

**Authors:** 


### THE ELECTROCUTION OF CZOLGOSZ.

From an account of the execution of Czolgosz contributed to the *New York Medical Record* by Dr. MacDonald, who was present, it appears that two electrical contacts were made occupying in all one minute and five seconds. In the first contact the electromotive pressure was maintained at 1,800 volts for seven seconds, then reduced to 300 volts for 23 seconds, increased to 1,800 volts for four seconds, and then reduced to 300 volts for 26 seconds, when it was broken. Why all this was done is not stated. It was conceded by all the witnesses, we are told, that conscious life was absolutely destroyed the instant the first contact was made. The poles had been connected with the head and leg, and the moment the circuit was completed by the turning of the switch the body was thrown into a state of extreme rigidity, every fibre of the muscular system appearing to be held in a marked condition of tonic spasm. At the same time consciousness, sensation and motion were apparently absolutely abolished. How under these circumstances the spectators arrived at any conclusion as to the state of consciousness of the victim we do not know. We have no doubt it was so. But it is quite clear that when all the muscles, the sole means by which expression can be given to any feeling, are in a state of tonic spasm, any such consciousness as a man might retain would have but little chance of giving evidence of its persistence.

